# Single-cell transcriptome sequencing reveals aberrantly activated inter-tumor cell signaling pathways in the development of clear cell renal cell carcinoma

**DOI:** 10.1186/s12967-023-04818-9

**Published:** 2024-01-08

**Authors:** Junfeng Zhang, Fuzhong Liu, Wenjia Guo, Xing Bi, Shuai Yuan, Fuerhaiti Shayiti, Ting Pan, Kailing Li, Peng Chen

**Affiliations:** 1 Department of Urology, Xinjiang Medical University Affiliated Tumor Hospital, Urumqi, China; 2 Cancer Institute, Xinjiang Medical University Affiliated Tumor Hospital, Urumqi, China; 3 Department of Urology, The Central Hospital of Enshi Tujia and Miao Autonomous Prefecture, No. 158 Wuyang Avenue, Enshi, 445000 Hubei China

**Keywords:** Clear cell renal cell carcinoma, Cell communication, Single-cell RNA sequencing, Cancer stem cells, SPP1 signaling pathway

## Abstract

**Background:**

Aberrant intracellular or intercellular signaling pathways are important mechanisms that contribute to the development and progression of cancer. However, the intercellular communication associated with the development of ccRCC is currently unknown. The purpose of this study was to examine the aberrant tumor cell-to-cell communication signals during the development of ccRCC.

**Methods:**

We conducted an analysis on the scRNA-seq data of 6 ccRCC and 6 normal kidney tissues. This analysis included sub clustering, CNV analysis, single-cell trajectory analysis, cell–cell communication analysis, and transcription factor analysis. Moreover, we performed validation tests on clinical samples using multiplex immunofluorescence.

**Results:**

This study identified eleven aberrantly activated intercellular signaling pathways in tumor clusters from ccRCC samples. Among these, two of the majors signaling molecules, MIF and SPP1, were mainly secreted by a subpopulation of cancer stem cells. This subpopulation demonstrated high expression levels of the cancer stem cell markers POU5F1 and CD44 (POU5F1^hi^CD44^hi^E.T), with the transcription factor POU5F1 regulating the expression of SPP1. Further research demonstrated that SPP1 binds to integrin receptors on the surface of target cells and promotes ccRCC development and progression by activating potential signaling mechanisms such as ILK and JAK/STAT.

**Conclusion:**

Aberrantly activated tumor intercellular signaling pathways promote the development and progression of ccRCC. The cancer stem cell subpopulation (POU5F1^hi^CD44^hi^E.T) promotes malignant transformation and the development of a malignant phenotype by releasing aberrant signaling molecules and interacting with other tumor cells.

**Supplementary Information:**

The online version contains supplementary material available at 10.1186/s12967-023-04818-9.

## Introduction

Renal cell carcinoma (RCC) is the deadliest tumor within the urological system, with clear cell renal cell carcinoma (ccRCC) being the most prevalent histological subtype [1]. Aberrant intercellular communication can result in uncontrolled cell proliferation, potentially leading to tumorigenesis and progression [2]. Nevertheless, the intercellular communication signals that facilitate ccRCC tumor cell growth in the setting of carcinogenesis are still not well understood.

Most RCC originates from renal tubular epithelial cells [3]. Currently, research on the driving factors of RCC primarily relies on genomic alterations, epigenetics, bulk RNA, and proteomic features [4]. For instance, in ccRCC, frequent biallelic loss of tumor suppressor genes on chromosome 3p, such as VHL, PBRM1, SETD2, and BAP1, can be detected [5]. Transcription factors such as HIF1A, MYC, and FOS are activated in ccRCC, promoting glycolytic metabolism, dedifferentiation, and growth [6, 7]. With the continuous advancement of single-cell transcriptome sequencing (scRNA-seq), we have the opportunity to gain in-depth insights into the complex cellular interactions within the tumor microenvironment (TME). ScRNA-seq enables us to thoroughly examine the disparities between tumor cells and normal cells, track the paths of cell growth and differentiation, and clarify the communication networks among cells [8, 9].

Intercellular communication involves transmitting messages from one cell to another through a medium to elicit a response [10]. Normal intercellular communication is crucial for preserving proper organizational function in multicellular organisms [11]. The transmission of signals between cells in the TME primarily occurs through direct cell-to-cell contact or by the action of paracrine signaling molecules such as cytokines, chemokines, growth factors, and protein hydrolases [12]. Cells in the TME communicate via receptors and ligands, creating a complex signaling network in cancer that is closely linked to a variety of behaviors, including cancer cell proliferation and immune escape [13, 14]. Disrupting or interfering with malignant signal transduction in intercellular communication is a crucial target for future cancer treatment strategies [15]. Given the current state of research and the significant role of intercellular communication signals in tumors, our study focused on inter-tumor cell signaling pathways in the development of ccRCC.

In this study, we analyzed scRNA-seq data from six normal kidney samples and six ccRCC samples. In the tumor cell clusters of ccRCC, we identified 11 aberrantly activated intercellular signaling pathways. Simultaneously, we discovered a subpopulation of cancer stem cells with the capability to secrete abnormal intercellular signaling substances. Moreover, we utilized multiplex immunofluorescence (mIF) to examine clinical samples, validating these findings.

## Methods

### Data and clinical sample collection

The scRNA-seq data used in this study were obtained from the GEO database (GSE159115: https://www.ncbi.nlm.nih.gov/geo/) [16]. The dataset includes 7 ccRCC tumor samples and 6 normal kidney tissue samples. For further analysis, we extracted high-quality scRNA-seq data from 6 ccRCC and 6 normal kidney tissue samples and discarded data from one low-quality ccRCC sample.

The collection of samples used in this study was approved by the Ethics Committee of the Affiliated Tumor Hospital of Xinjiang Medical University (K-2023028). We received written consent from participants that we informed before conducting the study. Tissue sections from four patients who underwent radical nephrectomy were procured from our hospital pathology department, including tumor and distal normal tissue. At least two pathologists verified the ccRCC pathotype and normal kidney tissue. The baseline characteristics of the clinical samples used in this study are summarized in Additional file 1: Table S1.

### Single-cell transcriptome sequencing data analysis

We used the Seurat R package (version 4.3.0) (https://satijalab.org/seurat/) [17] and followed the official user's guide for working with data. First, an initial quality control (QC) step was performed to filter high-quality cells, which included cells with 200–2500 RNA features and a mitochondrial gene proportion of less than 15%. We then normalized the data using the global scaling normalization method “LogNormalize” to ensure comparable RNA expression values in different cells. After identifying the genes with highly variable features in the dataset, we used principal component analysis (PCA) for dimensionality reduction. We selected the initial 20 principal components to capture major variants while still decreasing data dimensionality. The uniform manifold approximation and projection (UMAP) method is used in Seurat as a nonlinear reduction method to turn high-dimensional cell data into two-dimensional space. Thus, cells exhibiting similar expression patterns are categorized together, while those with dissimilar expression patterns can be segregated into different groups. Cell type annotation was carried out through a combination of automated and manual annotations to ensure accurate identification and annotation of each cell cluster.

### Single-cell copy-number variation analysis

To distinguish malignant cells in ccRCC samples, we assessed the copy number variation (CNV) of each cell in different chromosomal regions using the infercnv R software package (version 1.16.0) (https://github.com/broadinstitute/inferCNV) [18]. We used normal kidney epithelial cells as a reference to calculate CNV levels in the target cell clusters [19]. Subsequently, we generated a final CNV result file after normalizing the data using the expression levels of normal cells as a control. The CNV analysis was performed by running the “Infercnv::run” function with a cutoff value set to 0.1, and denoising and intermediate step plotting were enabled. To check for the presence of malignant cells, we filtered the InferCNV output based on “cell type” annotations that included “Magli.” Next, we extracted the CNV profiles of these epithelial cells for further analysis. Finally, we used Uphyloplot2 to construct phylogenetic trees depicting tumor evolution based on CNV data, categorizing the trees for each sample with consideration of tumor location and mutational status.

### Single-cell trajectory analysis

To study cell state transitions, we employed the Monocle R package (version 2.28.0) (http://cole-trapnell-lab.github.io/monocle-release/docs/) for trajectory analysis [20]. This involved selecting, sorting, and filtering genes from the scRNA-seq data, estimating size factors, and subsequently reducing dimensionality using the DDRTree algorithm. We visualized trajectories using cellular state plots and cell type maps and outlined distinct developmental trajectories for each cell type. To examine gene expression changes along these trajectories, we used color gradients to visualize specific genes. Additionally, heatmap analysis displayed the top five highly expressed genes within each group.

### Cell–cell communication analysis

We employed the CellChat R package (version 1.6.1) (https://github.com/sqjin/CellChat) to investigate intercellular communication and identify signaling molecules at the single-cell level [8]. Initially, we processed gene expression data to pinpoint highly expressed ligands and receptors within individual cell clusters. Next, we evaluated intercellular communication at the pathway level by computing communication probabilities for all ligand-receptor interactions associated with each signaling pathway. These probabilities were then aggregated to construct an intercellular communication network. Furthermore, to identify significant contributors within the cell–cell communication network, we calculated network centrality scores for each constituent and presented the findings visually. Additionally, we assessed and depicted signaling effects in both outgoing and incoming communication patterns. To comprehensively grasp how multiple cell clusters and signaling pathways synchronize their functions, we utilized CellChat’s pattern recognition approach to investigate diverse patterns of incoming and outgoing signal interactions among distinct cell clusters. Finally, to appraise aberrant signaling in tumor cells, we conducted a comparative analysis of the total count and strength of cell–cell interactions between tumor cells and epithelial cells.

### Single-cell transcription factor analysis

We used the SCENIC R package (version 1.3.1) (https://github.com/aertslab/SCENIC) to infer transcription factor regulatory networks and 4252 tumor cells from ccRCC samples [21]. We followed the official SCENIC guidelines and default parameters to standardize the analysis process. The SCENIC analysis consisted of three main steps. In the first step, the grnboost2 algorithm was employed to identify and filter genes co-expressed with transcription factors (TFs). The second step involved using RcisTarget to find significantly expressed target genes, then performing a significant motif enrichment analysis for each co-expressed module. In the third step, the transcriptional activity of each regulator was assessed using the AUCell algorithm. Cytoscape was used to portray the regulatory network connecting transcription factors and their target genes.

### Multiplex immunofluorescence staining

The levels of SPP1, POU5F1, CD44, JAK1, STAT3, and ILK proteins were detected through mIF. Sections were deparaffinized, underwent antigen retrieval, and were blocked with serum to prevent non-specific binding. Primary antibodies targeting the genes of interest were applied overnight at 4 °C, followed by washing and incubation with fluorescently labeled secondary antibodies for 1–2 h at room temperature. Nuclear staining was performed with appropriate dyes if necessary. Sections were then mounted and visualized using a fluorescence/confocal microscope.

### Statistical analysis

This study used R (version 4.3.1) for all statistical analyses. Two-tailed p-values were used for statistical significance. Differential gene expression analysis of different cell subpopulations was performed in the Seurat using the Wilcoxon rank sum test. The Monocle R package includes built-in t-tests and Wilcoxon rank sum tests for evaluating variations in gene expression. The SCENIC R package has a group of statistical methods, such as building co-expression networks, using hypergeometric and Fisher’s exact tests to find thematic enrichment, and using the AUCell algorithm to score transcription factor activity. p-values less than 0.05 were considered statistically significant (*, p < 0.05; **, p < 0.01; ***, p < 0.001).

## Results

### Establishment of a single-cell landscape for ccRCC and normal kidney tissues

**Fig. 1:**
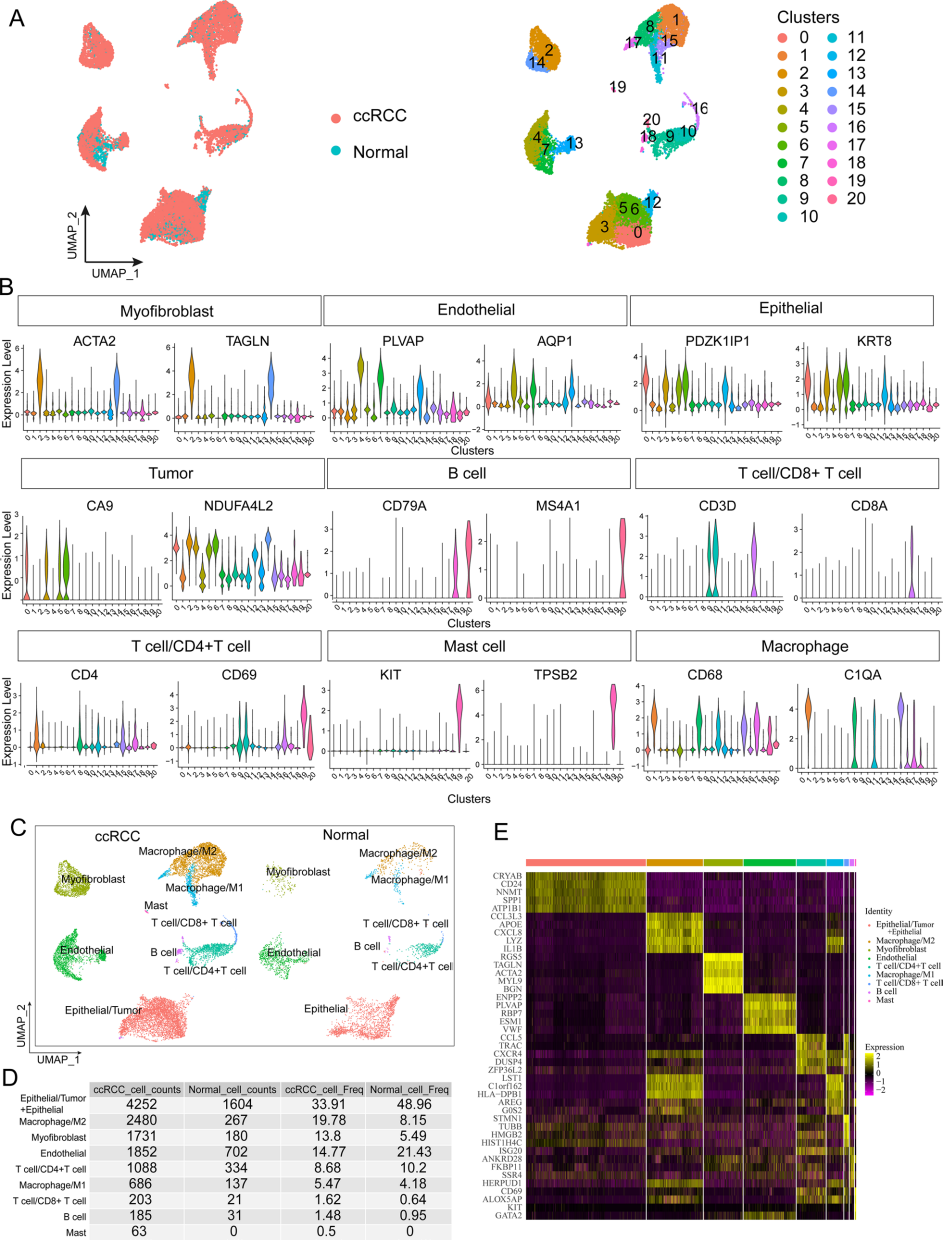
Single-cell atlas of ccRCC and normal kidney tissues. **A**. UMAP plots showing cell clustering, colored with ccRCC and normal kidney samples and with 21 cell clusters, respectively. **B**. Violin plots showing the expression of classical marker genes for cell types in different cell clusters. **C**. UMAP plots depicting the nine distinct cell types present in ccRCC and normal kidney samples. **D**. Number and proportion of different cell types. **E**. Heatmap showing the top 5 genes highly expressed in 9 cell types

To depict the single-cell atlas of ccRCC and normal kidney tissue, we conducted an in-depth analysis of scRNA-seq data from the GEO database (GSE159115). We extracted scRNA-seq data from 6 ccRCC samples and 6 normal kidney tissue samples from this dataset for analysis, and baseline characteristics of all samples have been described in the original study [16]. Following data quality control and filtering procedures, we constructed an atlas comprising 15,816 high-quality single cells and 23,541 genes and grouped these cells into 21 distinct clusters (Fig. 1A). We annotated the cell clusters based on the expression of classical cell marker genes [22] (Fig. 1B) and ultimately categorized the 21 cell clusters into 9 distinct cell types (Fig. 1C). There was an overlap in marker gene expression between tumor cells and epithelial cells in clusters 0, 3, 5, 6, and 12, leading to inaccurate distinguishing of tumor cells and epithelial cells in ccRCC tissues (Epithelial and Tumor in Fig. 1B). Therefore, we provisionally labeled clusters 0, 3, 5, 6, and 12 in the ccRCC samples as “Epithelial/Tumor”. Conversely, clusters 0, 3, 5, 6, and 12 in kidney tissue were confirmed to be epithelial cells (Fig. 1C). Based on the annotation results, the “Epithelial/Tumor” composition ratio was 4252 (33.91%) in ccRCC samples, while the epithelial cell composition ratio was 1604 (48.96%) in normal kidney tissue samples, totaling 5856 cells (Fig. 1D). The top 5 genes that are differentially expressed represent the transcriptional profiles of nine distinct cell types (Fig. 1E).

### Identification of tumor cells with two distinct transcriptional profiles in ccRCC samples

**Fig. 2:**
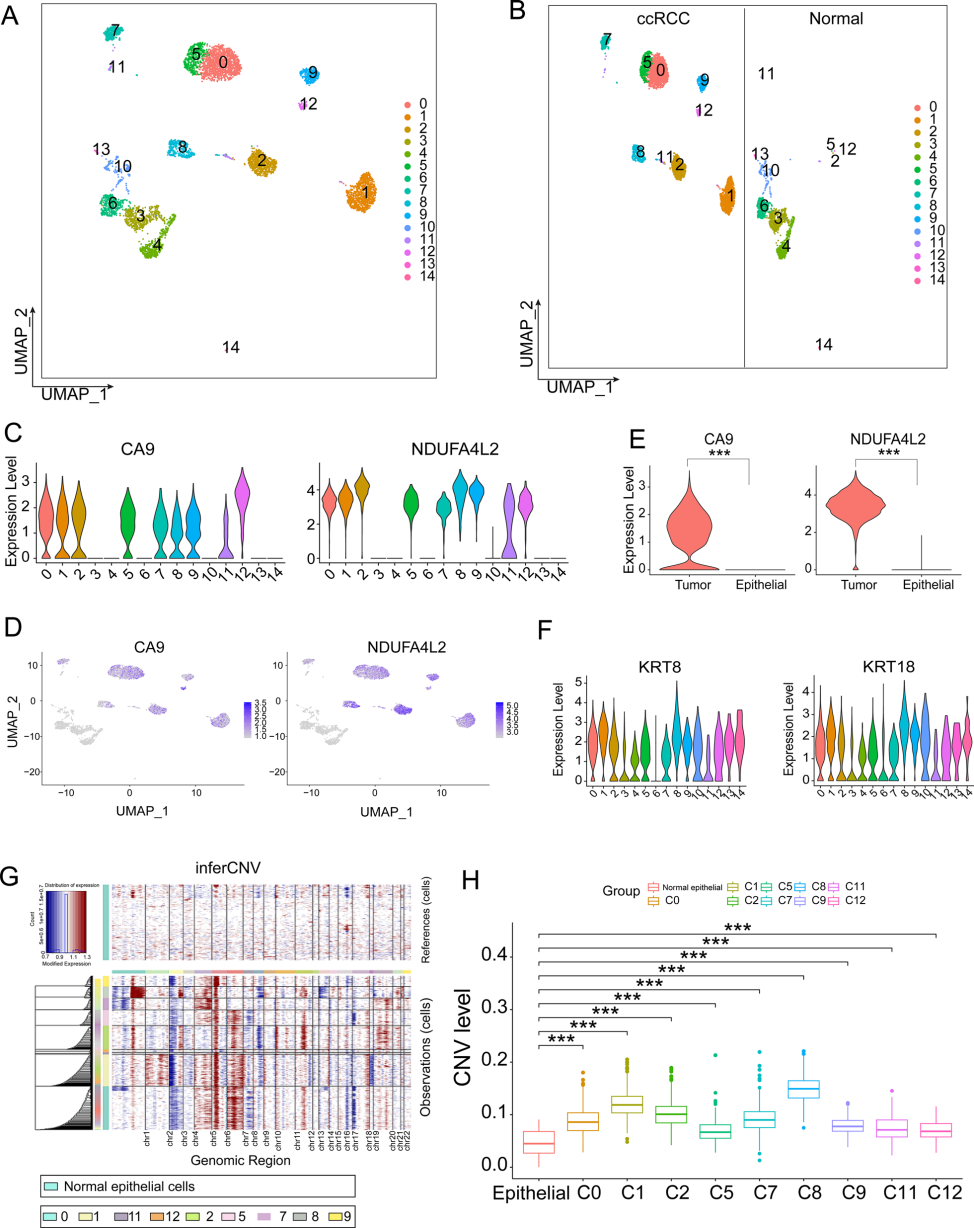
Distinguishing cell types of ccRCC samples. **A**. UMAP plot of subpopulation clustering analysis of 5856 cells. **B**. UMAP plot showing clusters of cells clustered in groups of ccRCC and normal samples. **C**. Violin plots depicting the expression of ccRCC tumor marker genes CA9 and NDUFA4L2. **D**. UMAP plots showing the different enrichment profiles of CA9 and NDUFA4L2 in the 15 subpopulations. **E**. Violin plot of CA9 and NDUFA4L2 expression levels significantly increased in tumor cells (tumor vs. epithelial, Mann–Whitney U test, ***p < 0.001). **F**. Violin plots showing the expression of the epithelial cell marker genes KRT8 and KRT18. **G**. Heatmap showing the results of CNV analysis. The upper heatmap represents the CNV profile of reference cells, while the lower heatmap represents the CNV profile of target cells. Red indicates CNV amplification, blue indicates CNV deletion and the depth of color represents the magnitude of CNV variation. **H**. Box plot illustrating the CNV levels across distinct cell clusters (Mann–Whitney U test, *** p < 0.001)

To accurately distinguish “Epithelial/Tumor” in ccRCC tissues, we performed in-depth analysis of 4252 “Epithelial/Tumor” cells in ccRCC tissues and 1604 epithelial cells in normal kidney tissues for a total of 5856 cells using four methods. Initially, we employed subpopulation clustering analysis to categorize the 5856 cells into 15 distinct subclusters (Fig. 2A). We identified 9 cell clusters in ccRCC samples and 10 cell clusters in normal samples. Notably, clusters 2, 5, 11, and 12 were observed in both types of samples, indicating that these cells share comparable transcriptional features. The reason for this is that one of the main goals of scRNA-seq is to identify populations of cells with similar transcriptome characteristics [23]. In contrast, clusters 3, 4, 6, 10, 13, and 14 were exclusive to normal samples, while clusters 0, 1, 7, 8, and 9 were exclusive to ccRCC samples (Fig. 2B). Thus, we annotated clusters 2, 5, 11, and 12 as well as clusters 3, 4, 6, 10, 13, and 14 derived from normal samples as epithelial cells.

Subsequently, based on the classical ccRCC marker genes [22], we found that clusters 0, 1, 2, 5, 7, 8, 9, 11 and 12 had significantly elevated levels of carbonic anhydrase 9 (CA9) and NADH dehydrogenase (ubiquinone) 1 alpha subcomplex, 4-like 2 (NDUFA4L2) gene expression (Fig. 2C–E). Conversely, other clusters (3, 4, 6, 10, 13 and 14) showed pronounced expression of epithelial cell markers while lacking tumor-specific markers, thus also demonstrating that these clusters were epithelial cells (Fig. 2F). High levels of CNVs are strongly linked to the development of cancer and can be used to identify possibly malignant cells based on their CNVs [24]. Using 1,604 epithelial cells from normal kidney tissue as a reference, we found significantly higher levels of CNV in clusters 0, 1, 2, 5, 7, 8, 9, 11, and 12 from ccRCC samples (Fig. 2G, H). Thus, clusters 2, 5, 11, and 12 as well as clusters 0, 1, 7, 8, and 9 from ccRCC samples were annotated as tumor cells.

**Fig. 3:**
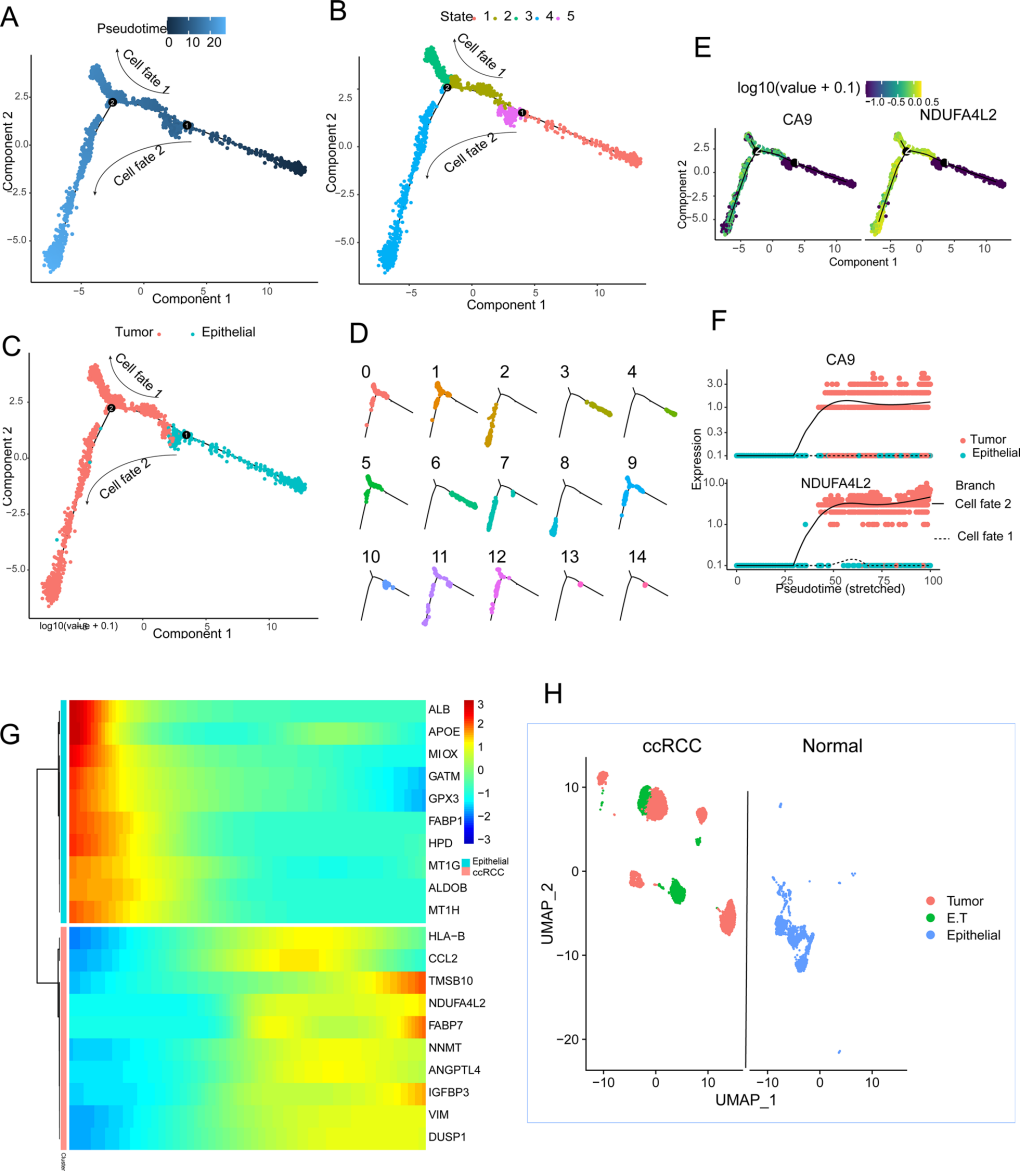
Distinguishing between cell types and cell states. **A**. Displaying the beginnings and endings of pseudo-time trajectories. The colors from dark to light represent the order of pseudo-time. **B.** Trajectory of cell differentiation showing five cell states. **C**. Differentiation trajectory showing the transformation of epithelial cells into tumor cells. **D**. Differentiation trajectories showing the positions of the 15 clusters. **E**. Differentiation trajectories demonstrating dynamic expression of the tumor marker genes CA9 and NDUFA4L2. **F.** Dot plots illustrating the dynamic expression of the tumor marker genes CA9 and NDUFA4L2 in cell fate 1 and cell fate 2. **G**. Heatmap depicting the highly expressed T0P10 genes in tumor cells and epithelial cells. **H**. UMAP plot of final annotated cell types

Finally, we further validated the annotation results using scRNA-seq trajectory analysis. We constructed differentiation trajectories for 15 cell clusters and identified five distinct cell states (Fig. 3A, B). The process of transforming epithelial cells into malignant cells can be observed through the developmental trajectory consisting of cell fate 1 and cell fate 2 (Fig. 3C). The trajectories of differentiation that consist of 6 cell clusters (3, 4, 6, 10, 13, 14) mainly represent the trajectories of epithelial cell differentiation and cell states 1, 5. The trajectories of differentiation that consist of the remaining 9 cell clusters mainly represent the trajectories of tumor cell differentiation and cell states 2, 3, 4 (Fig. 3D). Dynamic expression changes were observed for CA9 and NDUFA4L2 along consistent trajectories in the differentiation from epithelial cells to malignant cells (Fig. 3E, F). In tumor cells, we observed activation of genes associated with cell proliferation (TMSB10, IGFBP3), migration (CCL2), signal transduction (DUSP1), and other behaviors (Fig. 3G).

As tumor clusters 2, 5, 11, and 12 of ccRCC retained the transcriptional characteristics of epithelial cells, we defined them as epithelial tumor cells (E.T) and labeled them C2-E.T, C5-E.T, C11-E.T, and C12-E.T (Fig. 3H). In terms of the transcriptional characterization of individual cells, these E.T cells exhibit an intermediate transcriptional state between epithelial and malignant cells. Cancers that originate from epithelial cells retain their epithelial cell characteristics during the early stages of development [25]. We speculate that these E.T clusters may play a crucial role in the early stages of tumor development, preserving abnormal information associated with tumorigenesis.

### Construction of an intercellular communication atlas for the 15 cell clusters

**Fig. 4:**
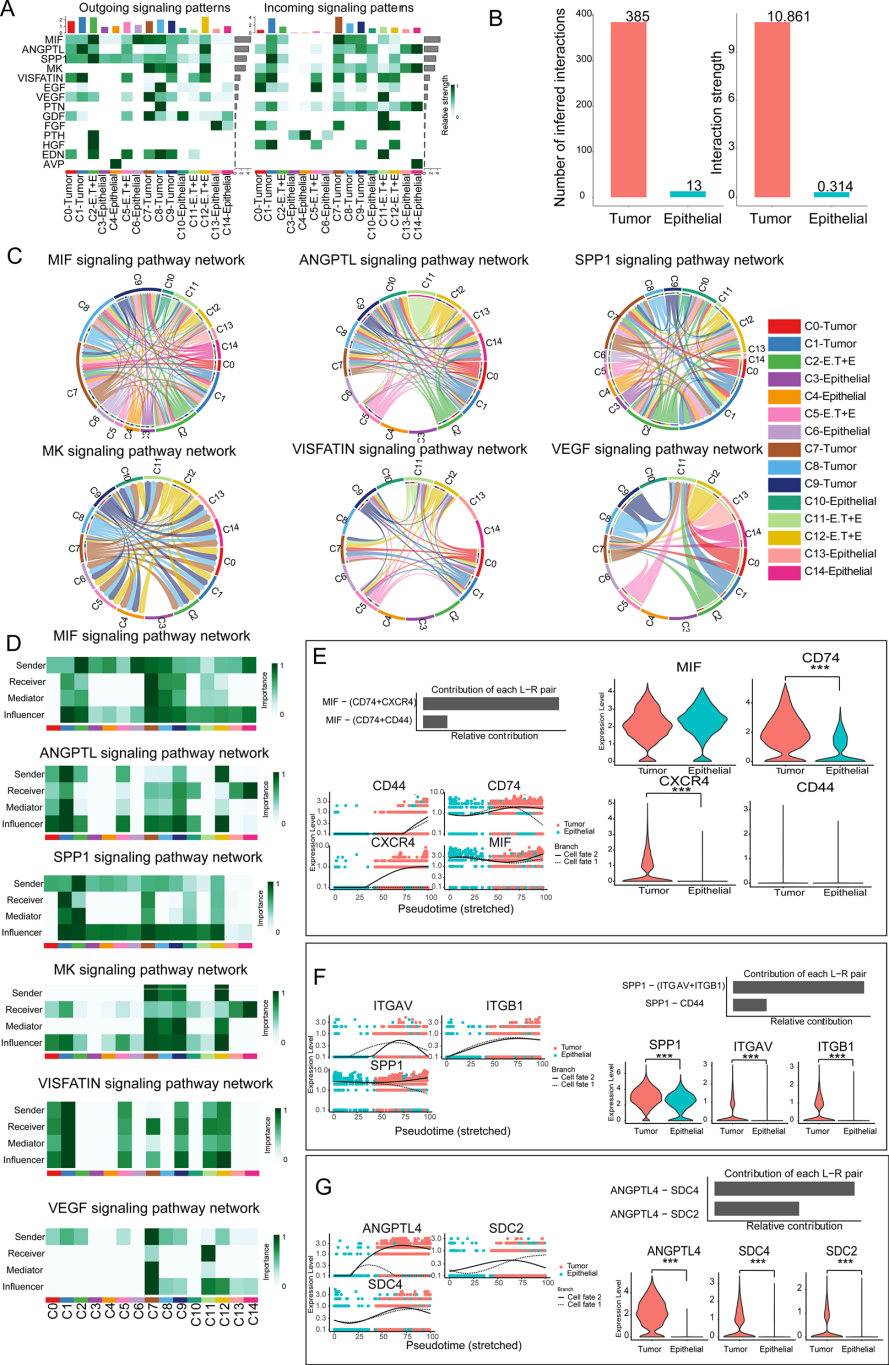
Intercellular communication network atlas of 15 cell clusters. **A**. Heatmap showing the top 14 signals in the 15 cell clusters with the largest contribution of outgoing or incoming signals. **B**. Bar charts illustrating the quantity and intensity of intercellular communication. **C**. Chord diagrams of six signaling pathways showing complex interactions between cell populations. **D**. Heatmaps depicting network centrality scores for the six signaling. **E**–**G**. Bar graphs depicting ligand-receptor pairs mediating the communication of the MIF, SPP1, and ANGPTL signaling; dot plots of the dynamic expression changes of receptor-ligand pairs associated with the MIF, SPP1, and ANGPTL signaling in the pseudo temporal trajectory; violin plots depicting the differential expression of MIF, SPP1, and ANGPTL signaling L-R pair genes between tumor cells and epithelial cells (Mann–Whitney U test, ***p < 0.001). E.T + E indicating that the cluster consists of E.T cells and epithelial cells

We utilized the CellChat R package [8] to construct a cell–cell communication atlas of 15 clusters, consisting of a total of 5856 cells from both normal samples and ccRCC samples. Firstly, we identified the top 14 signaling pathways contributing significantly to outgoing and incoming signals among the 15 cell clusters. Among these pathways, the top 5 based on their contribution values are MIF, ANGPTL, SPP1, MK, and VISFATIN (Fig. 4A). The intercellular communication network of tumor cells exhibits a significantly higher number of interactions and interaction strength compared to epithelial cells (Fig. 4B). VEGF-targeted therapy is the first-line treatment for advanced RCC [26]. We identified complex interactions between clusters through the top 5 signaling pathways as well as the VEGF signaling pathway (Fig. 4C). Continuing, we identified the key senders, receivers, intermediaries, and influencers within these six signaling networks among the cells by calculating several network centrality scores for each cell group (Fig. 4D).

In addition, we identified MIF-(CD74 + CXCR4) and MIF-(CD74 + CD44) as the most contributing ligand-receptor pairs (L-R pairs) to MIF signaling pathway. The expression of the receptors CXCR4 and CD74 was markedly increased in tumor cells, suggesting activation of the MIF signaling pathway in tumors (Fig. 4E). We discovered L-R pairs in both the SPP1 and ANGPTL signaling pathways, and determined that the related genes were considerably elevated in tumor cells (Fig. 4F, G). Taken together, this part of our results constructs a general atlas of cell–cell communication between 15 cell clusters.

### Discovering physiological intercellular signaling pathways between epithelial cells and aberrant intercellular signaling pathways between tumor cells

**Fig. 5:**
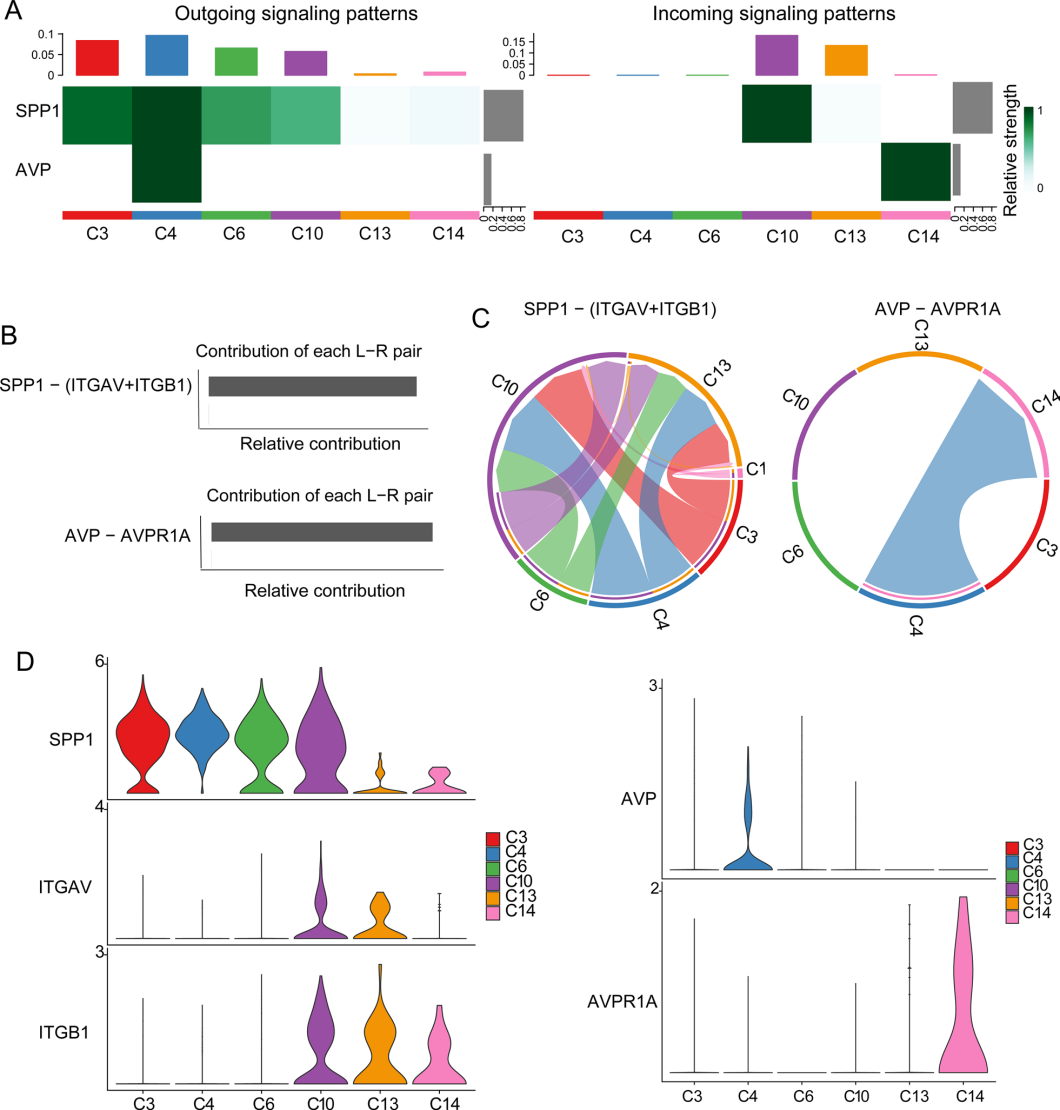
Cell–cell communication between six epithelial cell cluster. **A**. Heatmap showing two physiological signaling pathways in six clusters. **B**. Bar graphs showing L-R pairs that mediate communication in the SPP1and AVP signaling pathway. **C**. Chord diagrams of SPP1 and AVP signaling pathway-mediated interactions between six epithelial cell clusters. **D**. Violin plots illustrating the gene expression of receptor-ligand pairs of the SPP1 and AVP signaling pathway

Intercellular communication between normal epithelial cells is essential for maintaining physiological functions [27]. We identified two major physiological intercellular signaling pathways, SPP1 and AVP, in six key epithelial cell clusters (Fig. 5A). The communication between these cell clusters is primarily mediated by the L-R pairs SPP1-(ITGAV + ITGB1) and AVP-AVPR1A (Fig. 5B, C). Specific signaling molecules (SPP1, AVP) are secreted by clusters of cells, while clusters expressing corresponding receptors (ITGAV + ITGB1, ACPR1A) serve as target cells (Fig. 5D). The interaction of these signaling molecules with receptors on the surface of target cells establishes the communication pathways between different cell clusters.

**Fig. 6:**
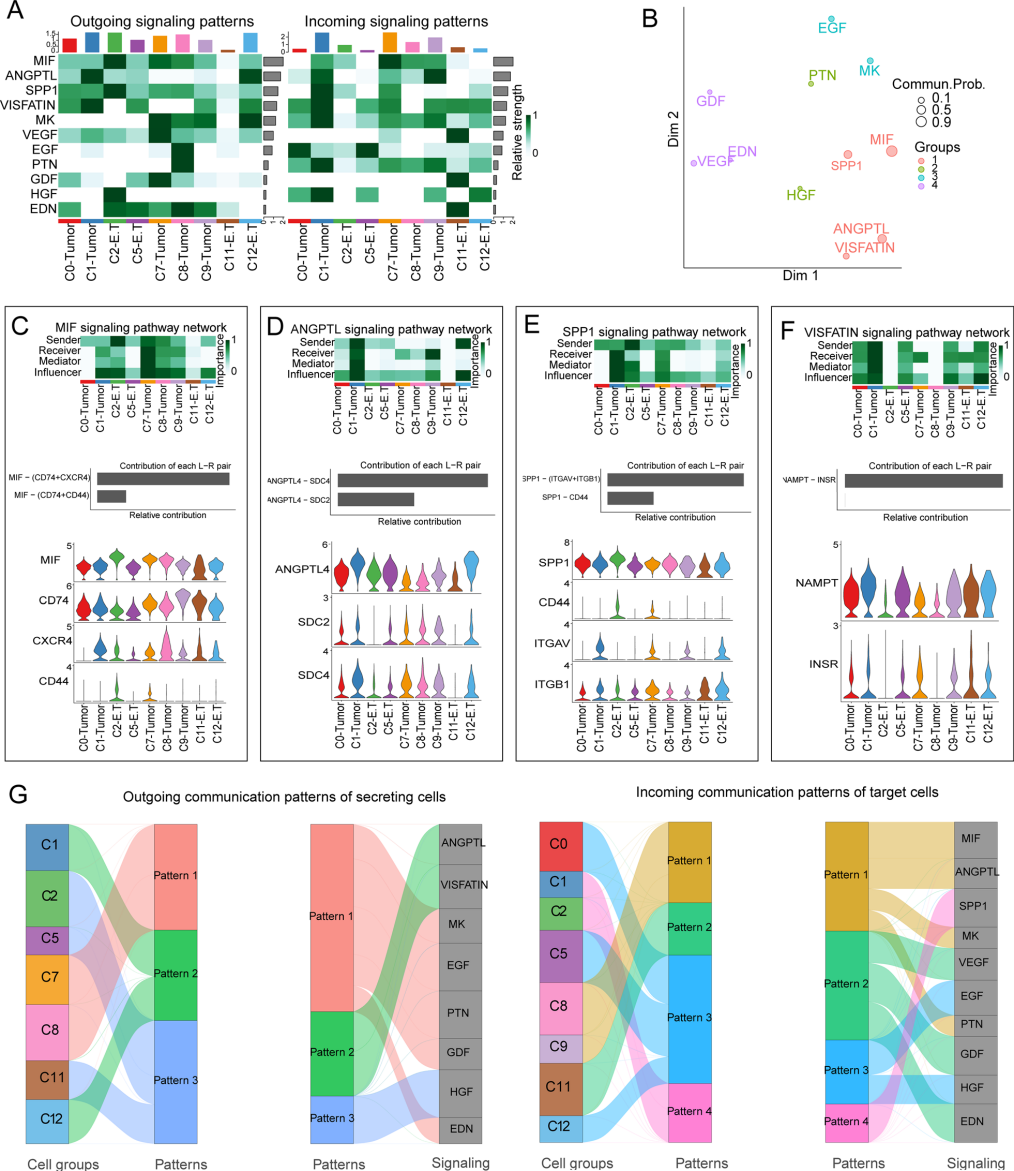
Cell–cell communication between the 9 tumor cell clusters in ccRCC tissues. **A**. The heatmap showing the top 11 signals in the 9 tumor cell clusters with the largest contribution of outgoing or incoming signals. **B**. Dot plot showing 11 signals divided into 4 groups of signal modules based on functional similarity. **C**. Heatmap depicting network centrality scores for the MIF signaling; bar graph showing L-R pairs that mediate communication in the MIF signaling pathway; violin plots illustrating the expression of receptor-ligand pairs of the MIF signaling pathway. **D**–**F**. Figures displaying network centrality scores (heatmaps), ligand-receptor pairs mediating communication (bar graphs), and expression of receptor-ligand pairs (violin plots) for ANGPTL, SPP1, and VISFATIN signaling pathways. **G**. River diagrams showing the communication patterns that coordinate secretory cell clusters and outgoing signaling pathways, as well as those that coordinate target cell clusters and incoming signaling pathways

Next, we identified eleven aberrantly activated intercellular signaling pathways in nine tumor clusters from ccRCC samples. The strongest signaling of MIF, SPP1, and HGF was observed in C2-E.T, while the strongest signaling of ANGPTL and MK was observed in the C12-E.T cluster (Fig. 6A). The eleven signaling pathways were classified into distinct functional groups: GROUP1 represents inflammation-related signals, GROUP2 represents angiogenesis signals, GROUP3 represents cell proliferation signals, and GROUP4 represents signals associated with cell differentiation and survival (Fig. 6B). We focused on studying the TOP4 signaling pathways (MIF, ANGPTL, SPP1, and VISFATIN), which have the greatest contribution to intercellular signaling between cell clusters. For the MIF signaling pathway, the primary sender of MIF signals is the C2-E.T cluster, while the most crucial receiver of signals is the C7-Tumor cluster. The transmission of MIF signals primarily occurs through L-R pairs MIF − (CD74 + CXCR4) and MIF − (CD74 + CD44). Except for CD44, which was only expressed in the C2-E.T and C7-Tumor cell clusters, all receptor and ligand genes were broadly expressed among these nine cell clusters (Fig. 6C). We examined the ANGPTL, SPP1, and VISFATIN signaling pathways using the same analytical approach (Fig. 6D–F). Notably, the C2-E.T cell clusters were also the foremost senders of SPP1 signals, while the primary influencers and receivers of these signals were the C1-Tumor clusters (heatmap in Fig. 6E).

Communication between cells is essential for carrying out complex biological functions, the coordinated between different cell populations and signaling pathways makes these functions possible [28]. We identified three outgoing communication modes for secretory cells and four incoming communication modes for target cells to coordinate signaling. For example, in C7 and C8 tumor clusters, outgoing pattern 1 coordinates signals like MK, EGF, PTN, GDF, and EDN. In C8 and C9 tumor clusters, incoming pattern 1 coordinates signals like MIF, ANGPTL, MK, and PTN (Fig. 6G).

Taken together, the intercellular communication signals of tumor cell cluster interactions are much more abundant and complex.

### Transcription factor analysis reveals POU5F1 targeting regulation of SPP1 in the C2-E.T cluster

**Fig. 7:**
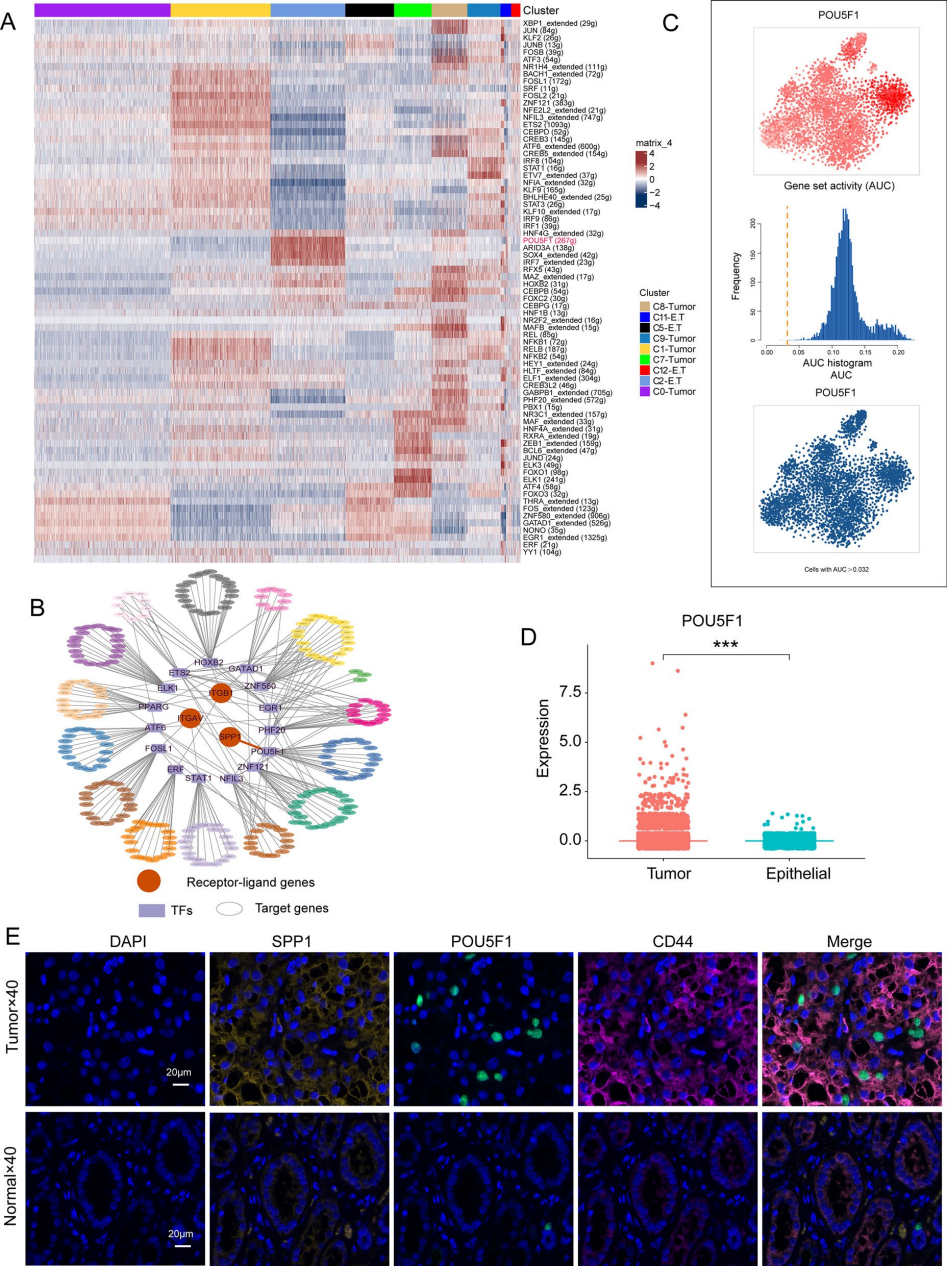
Transcription factor analysis of 4252 cells from 9 tumor cell clusters. **A**. Heatmap showing transcription factor activity enriched in different cell clusters. **B**. Regulatory network of transcription factors with targeted regulatory effects on genes by SPP1-(ITGAV +  + ITGB1) L-R pairs. **C**. AUCell quantification of POU5F1 transcription factor activity. **D**. Box plot illustrating significantly higher expression of POU5F1 in tumor cells than in epithelial cells (Mann–Whitney U test, ***p < 0.001). **E**. Multiplex immunofluorescence staining in ccRCC samples and in normal kidney tissues for verification of SPP1, POU5F1 and CD44 gene expression. Renal tubular epithelial cells arranged in a ring-like pattern in normal kidney tissue

We utilized the SCENIC R package (v1.3.1) to analyze 4252 cells from 9 tumor clusters in ccRCC samples for the identification of TFs [21, 29]. In the C2-E.T cluster, we identified five highly transcriptionally active TFs (HNF4G, POU5F1, ARID3A, SOX4, and IRF7) (Fig. 7A). TF regulatory network analysis further revealed 15 TFs targeting the SPP1-(ITGAV + ITGB1) L-R pair, with POU5F1 identified as a key regulator of SPP1 (Fig. 7B). Subsequently, we quantified the activity of POU5F1 using AUCell and observed an enriched AUC peak in the C2-E.T cluster (Fig. 7C). The C2-E.T cluster exhibited concurrent high expression of POU5F1 and CD44, recognized markers of cancer stem cells (CSCs) [30, 31], indicating the presence of CSCs attributes in the C2-E.T cluster. Hence, the C2-E.T cluster was designated as the POU5F1^hi^CD44^hi^E.T subpopulation. Compared to epithelial cells, POU5F1 expression was significantly higher in tumor cells (Fig. 7D). Finally, strong fluorescent signals of SPP1, POU5F1, and CD44 were observed in ccRCC clinical samples, confirming the above phenomenon at the protein expression level (Fig. 7E). Together, we identified a subpopulation of CSCs, and a potential transcriptional regulatory mechanism for the SPP1 signaling molecule secreted by this subpopulation.

### The inter-tumor cell SPP1 signaling pathway promotes malignant phenotypes

**Fig. 8:**
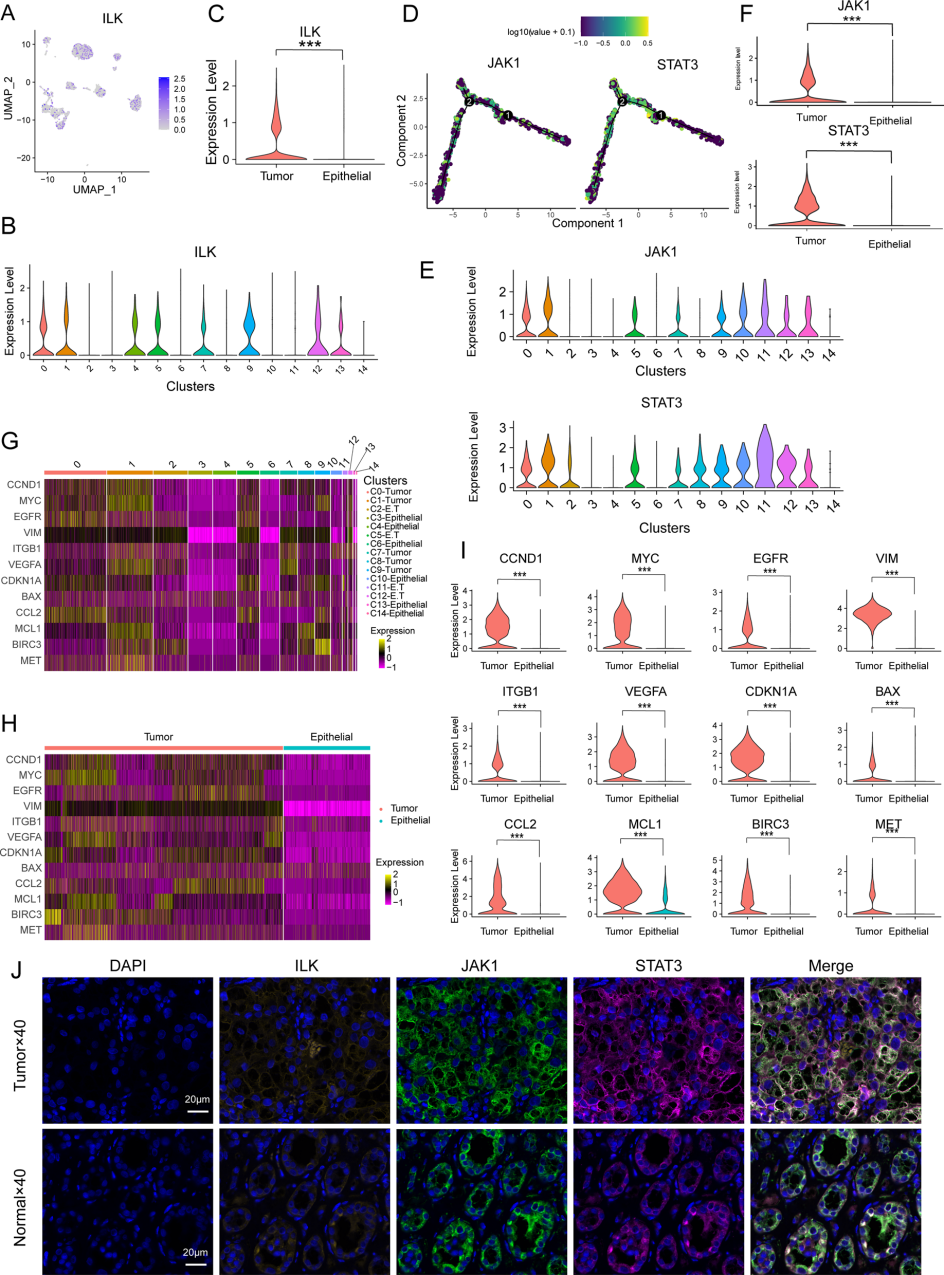
Effect of the SPP1 signaling pathway on the malignant behavior of tumor cells. **A**, **B**. UMAP plot and violin plot showing the expression of ILK in 15 clusters. **C**. Violin plot illustrating the expression difference of ILK between tumor cells and epithelial cells (Mann–Whitney U test, ***p < 0.001). **D**. Pseudo-time trajectory showing changes in dynamic expression of JAK1 and STAT3. **E**. Violin plots of JAK1 and STAT3 enrichment in 15 cell clusters. **F**. Violin plots showing significant overexpression of the JAK1 and STAT3 genes in tumor cells (Mann–Whitney U test, ***p < 0.001). **G**. Heatmap illustrating gene expression of markers associated with malignant phenotypes in clusters. **H**. Heatmap showing gene expression of markers associated with malignant phenotypes in tumor cells and epithelial cells. **I**. Violin plots comparing gene expression markers linked to malignant phenotypes in tumor cells and epithelial cells (Mann–Whitney U test, ***p < 0.001). **J**. Multiplex immunofluorescence staining in ccRCC samples and in normal kidney tissues for verification of ILK, JAK1, and STAT3 gene expression

Following secretion by cells, SPP1 signaling molecules bind to the integrin receptor (αβ1, encoded by ITGAV and ITGB1) on the surface of target cells, facilitating the transmission of extracellular signals into the intracellular environment [32]. The transduction of signals leads to target cell polarity, gene expression, cell survival, and proliferation [33]. To investigate the role of SPP1 signaling on tumor cells, we analyzed 4252 tumor cells from ccRCC samples and 1604 epithelial cells from normal samples. The signal transduction mediated by integrins typically requires the activation of adhesion kinases such as focal adhesion kinase (FAK), SRC family kinases, and integrin-linked kinase (ILK) [34, 35]. We observed a markedly elevated expression of ILK in tumor clusters when compared to epithelial cells (Fig. 8A–C). ILK, as a pro-proliferative protein kinase, serves as a central hub for integrin-mediated intracellular signaling, activating downstream pathways such as PI3K/AKT, Wnt/β-catenin, MAPK, and others [35, 36]. We observed significant overexpression of JAK1 and STAT3 in tumor cells, which are pivotal members of the JAK/STAT pathway (Fig. 8D–F).

Subsequently, we investigated marker genes associated with the biological behaviors of cancer cells and identified 12 marker genes enriched in clusters of tumor cells (Fig. 8G). These marker genes represent multiple malignant phenotypes of cancer, including tumorigenesis (EGFR, MYC), cell invasion (ITGB1 and VIM), and angiogenesis (VEGFA), among others. Moreover, these 12 marker genes were significantly highly expressed in tumor cells (Fig. 8H, I). Multiplex immunofluorescence staining further demonstrated elevated expression of ILK, JAK1, and STAT3 in ccRCC samples (Fig. 8J). These results indicate that the SPP1 signaling pathway may promote the development of a malignant phenotype by activating intracellular signals such as ILK, JAK/STAT in target cells.

## Discussion

In this study, we depicted the complex interactions mediated by intercellular signaling pathways between tumor clusters in ccRCC samples. Furthermore, we identified a subpopulation of CSCs and elucidated their critical role in driving malignant transformation. These results advance our knowledge of the development of ccRCC and offer theoretical justification for novel therapeutic approaches that focus on CSC subpopulations and intercellular pro-cancer signaling pathways.

Cancers originating from epithelial cells maintain some epithelial cell characteristics [25]. The transformation process of epithelial cells into malignant cells exhibits a continuous, dynamic, and multistage progression. Epithelial-mesenchymal transition (EMT) is a critical feature of cancer development and metastasis, and cancer cells in the partial EMT (p-EMT) stage can express both epithelial and mesenchymal markers [23]. The discovery of E.T. clusters lay the foundation for investigating crucial intercellular signaling events during the malignant transformation and early stages of onset in ccRCC. However, single-cell trajectory analysis did not definitively reveal the continuous developmental trajectory of the E.T. clusters. This may be attributed to the transient state of E.T. clusters being closer to the transient state of malignant cells, resulting in a merging of the trajectories between E.T. clusters and malignant cells.

Intercellular communication is of paramount importance in coordinating the behavior of individual cells during the development of an organism [37]. In epithelial cells, we identified the physiological pathways of SPP1 and AVP, where SPP1 inhibits aspects of calcium oxalate crystallization, and the AVP pathway is essential for regulating fluid homeostasis and maintaining blood pressure [38, 39]. The uncontrolled proliferation resulting from aberrant regulation of cellular signaling represents a critical mechanism underlying the onset of cancer [40]. On the contrary, we identified 11 significantly active signaling pathways in tumor cell clusters, including the SPP1 signaling pathway. Signaling molecules such as MIF and SPP1 are predominantly delivered by the C2-E.T cluster and represent main the pro-cancer signals between cells in the early stages of tumorigenesis. Previous research has demonstrated that SPP1 serves an important function in cellular signaling, facilitation of angiogenesis, and evasion of immune responses across various cancer types [41, 42]. Macrophage migration inhibitory factor (MIF) is a versatile cytokine that stimulates pro-inflammatory, chemotactic, and growth responses within cells [43]. MIF promotes cancer cell proliferation and metastasis by activating various signaling pathways, including ERK, MAPK, and Akt [44].

By recognizing specific DNA sequences, TFs control chromatin and transcription, which in turn controls processes related to cell fate determination, developmental patterns, and control of specific pathways [45]. The specific expression of TFs typically corresponds to their respective specialized functions [46]. We observed that the transcription factor POU5F1 exhibited high transcriptional activity in the C2-E.T cluster and targeted the SPP1 gene. The POU5F1 gene encodes Octamer-Binding Transcription Factor 4 (Oct4), a classical marker of CSCs that assists in maintaining their self-renewal capacity [30]. However, overexpression or aberrant regulation of POU5F1 is associated with tumorigenesis and progression, potentially instigating uncontrolled cell division and contributing to the maintenance of CSCs [47]. The activity of POU5F1 is regulated by various factors, including epigenetic modifications, IGF-IR/IRS-1/PI3K/AKT/GSK3b cascade signaling, and interactions with other transcription factors [48]. This discovery indicates that POU5F1 regulates the transcription of the SPP1 gene, promoting the secretion of SPP1. Furthermore, CD44 serves as a cell surface marker for CSCs and contributes significantly to the maintenance of stemness and regulation of cancer stem cells [31]. Accordingly, the study identified a subpopulation of cancer stem cells (POU5F1^hi^CD44^hi^ E.T.) that send early critical pro-cancer signals. Further investigation revealed that SPP1 signaling from this subgroup promotes malignant phenotypes through potential signal transduction mechanisms such as activation of ILK and JAK/STAT.

CSCs possess remarkable differentiation potential, the ability to proliferate indefinitely, and the capacity to regenerate themselves [49]. CSCs contribute significantly to tumorigenesis, metastasis, recurrence, heterogeneity, drug resistance, and immune evasion, and are a significant cause of failed tumor treatments [50]. Targeting cancer stem cells may decrease cancer recurrence and metastasis, enhance the comprehensiveness and long-term effectiveness of treatment, and enhance patient prognosis. Several therapeutic strategies exist for targeting CSCs, such as inhibiting signaling pathways associated with CSCs, DNA damage repair, ALDH targeting [51]. For instance, numerous intracellular signaling pathways, including Notch, JAK/STAT, PI3K/AKT/mTOR, have significant involvement in sustaining the activity of CSCs [52]. Humans have developed many drugs against these pathways and targets. However, therapies targeting CSCs are still in their infancy and are limited by difficulties such as lack of specific markers and high side effects. In addition to developing novel medications for newly identified essential targets, drug repurposing of older drugs is an important strategy for optimizing therapeutic regimens [53]. For instance, in the cases of psoriasis [54], certain viral cancers [55] and the initial outbreak of COVID-19 [56, 57], all of which faced the dilemma of having no drugs available for treatment or poor results from existing treatment options, drug repurposing became an effective alternative.

Blocking or interfering with intercellular communication shows tremendous potential as an anti-cancer strategy [15]. For instance, VEGF-targeted therapy is employed for the treatment of a range of cancer types, such as colorectal cancer, kidney cancer, and non-small cell lung cancer [58]. MIF and SPP1 play critical roles in various diseases, including cancer, and present promising therapeutic targets [59, 60]. Despite this potential, anti-cancer drugs that target MIF (Imalumab: NCT01765790) and SPP1(BET inhibitors)[42] are still in the exploratory or early clinical trial phase. Prior to moving these therapeutic strategies to the clinical stage, multiple challenges must first be addressed. These obstacles include theoretical guidance for basic research, clinical trial design, and drug safety.

This study still has several limitations. First, the relatively small sample size may limit the generalizability of the study results. Secondly, functional experiments are required to confirm the precise roles and mechanisms of aberrant intercellular signals in tumorigenesis. Additionally, single-cell RNA sequencing data lacks cellular spatial information, making it challenging to provide comprehensive and representative spatial details. As part of future research, functional experiments should be planned to confirm the exact roles and mechanisms of abnormal intercellular signals in the development of ccRCC. At the same time, it is very important to learn more about CSCs in ccRCC, including finding specific markers and ways to stop them from working. Multi-omics analyses, including spatial transcriptomics and the joint analysis of large cohort samples, hold the potential to provide more comprehensive information.

## Conclusion

In summary, this study identified 11 aberrantly activated inter-tumor cell signaling pathways in the development of ccRCC. Moreover, a CSCs subpopulation (POU5F1^hi^CD44^hi^E.T) was identified. This subpopulation interacts with other tumor cells through the secretion of the aberrant signaling molecule, which promotes malignant transformation and a malignant phenotype. The research provides valuable insights into the mechanisms of ccRCC development from the perspective of intercellular communication. However, further research is needed to validate these findings and explore more precise CSCs markers, strategies to block the function of signaling substances secreted by CSCs, or even to eliminate CSCs.

### Supplementary Information

**Additional file 1: Table S1.** Baseline characteristics of clinical samples

## Data Availability

All data generated or analyzed during this study were included in this article's methods section. Other data that support the findings of this study are available from the corresponding author upon reasonable request.

## Abbreviations

RCC: Renal cell carcinoma
ccRCC: Clear cell renal cell carcinoma
ScRNA-seq: Single-cell RNA sequencing
CNV: Copy number variations
TFs: Transcription factors
E.T: Epithelial tumor cells
CSCs: Cancer stem cells
CA9: Carbonic anhydrase 9
NDUFA4L2: NADH dehydrogenase (ubiquinone) 1 alpha subcomplex, 4-like 2
EMT: Epithelial-mesenchymal transition
